# Reducing group delay spread using uniform long-period gratings

**DOI:** 10.1038/s41598-018-21609-1

**Published:** 2018-03-01

**Authors:** Huiyuan Liu, He Wen, Bin Huang, Rodrigo Amezcua Correa, Pierre Sillard, Haoshuo Chen, Zhihong Li, Guifang Li

**Affiliations:** 1 CREOL, The College of Optics & Photonics, University of Central Florida, Orlando, FL 32816 USA; 2 The College of Precision Instruments and Opto-elctronic Engineering, Tianjin University, Tianjin, 300072 P.R. China; 3 Prysmian Group, Parc des Industries Artois Flandres, Haisnes, 62092 France; 4 Bell Laboratories, Alcatel-Lucent, 791 Holmdel Rd, Holmdel, NJ 07733 USA; 5 Futurewei Technologies Inc, 2330 Central Expressway,, Santa Clara, CA 95050 USA

## Abstract

Despite the promise of an orders-of-magnitude increase in transmission capacity, practical implementation of mode-division multiplexing faces a number of challenges. The most important among them is the complexity of digital signal processing (DSP) for compensating mode crosstalk and modal dispersion. The most promising method proposed so far for reducing this DSP complexity is strong mode coupling. We propose and demonstrate, for the first time, a method of inducing strong mode coupling and reducing group delay spread using uniform long-period gratings (LPGs). Even though the LPGs have a fixed grating period, mode coupling is effective among all mode groups and over a broad wavelength range. Both insertion loss and mode-dependent loss can be significantly reduced by optimizing the index profile of and the number of modes supported by the fiber in which the LPG is applied.

## Introduction

As single-mode fiber-optic communication systems approach their capacity limit, space-division multiplexing (SDM) has attracted significant attention in recent years^1,2^. SDM uses the spatial degrees of freedom to increase the number of parallel independent data channels in an optical fiber. For example, in mode-division multiplexed (MDM) transmission, each spatial mode of a few-mode fiber (FMF) carries independent information. Because of random, distributed mode crosstalk and modal dispersion, multiple-input-multiple-output (MIMO) digital signal processing (DSP) is required to recover the independent data channels at the receiver^3,4^. The complexity of MIMO DSP scales with the group delay spread (GDS), which generally increases linearly with the transmission distance^5^. High MIMO complexity not only leads to high cost but also high power consumption. As both passive and active devices for MDM such as mode (de)multiplexers and few-mode amplifiers mature, MIMO DSP complexity presents itself as the most challenging obstacle to the future deployment of MDM systems.

So far, several methods for reducing the GDS have been proposed. They include optimizing fiber index profiles^6^ to reduce differential modal group delay, akin to dispersion-shifted fibers, and modal group delay compensation^7^, akin to chromatic dispersion compensation in single-mode fibers (SMFs). However, both of these methods are effective only for a small number of modes^8^. The most promising method proposed so far is strong mode coupling^9,10^. When modes are weakly coupled, the GDS increases linearly with the transmission distance. When modes in a FMF are strongly coupled, the GDS increases with the square root of the transmission distance. This is because each MDM signal would have a nearly equal probability of traveling on different modes averaged over the transmission link. A straightforward approach of introducing strongly mode crosstalk is to use a scrambler sandwiched between a mode multiplexer (MUX) and a mode demultiplexer (DMUX)^11^. This approach not only is expensive but also likely incurs losses much higher than what is required as described below.

Weak mode coupling occurs randomly and parasitically in FMFs, but strong mode coupling must be introduced intentionally. A long-period grating (LPG) is one of the most effective structures to promote strong mode coupling. Since mode coupling induced by LPGs is a coherent, phase-matched process, a different grating is required for each pair of modes (or mode groups)^9,12^. An intrinsic problem with this approach is that any LPG used for mode coupling can also couple the highest-order guided mode (group) to a cladding mode in a phase-matched manner due to the high density of cladding modes. In addition to this intrinsic loss, each LPG also has extrinsic loss due to imperfections in the grating. Therefore, as the dimension of MDM increases, so does the number of LPGs. A large number of LPGs obviously adds complexity and cost, but a more serious drawback is the loss accumulated by each LPG. Because loss has the most direct impact on the capacity of a communication channel and even 0.01 dB/km of loss reduction is being sought for SMFs, the LPG used to induce strong mode coupling must also introduce extremely low losses, preferably below 0.1 dB, to ensure that the transmission capacity of an MDM system is competitive with parallel SMF transmission systems.

In this paper, we present a practical approach to achieving low-loss strong mode coupling using LPGs by reducing both the number of LPGs and the intrinsic loss of each LPG due to coupling to cladding modes. This approach is based on applying the LPG on properly designed graded-index (GRIN) FMFs. First, we show that it is possible to use only one uniform LPG with a fixed grating period to introduce efficient coupling not only among all modes but also over a broad range of wavelengths. The use of only one grating minimizes the extrinsic loss to the largest extent possible. Furthermore, through simulations, we also demonstrate the reduction of intrinsic loss and mode-dependent loss (MDL) by optimizing the index profile of and the number of modes supported by the fiber in which the LPG is applied.

## FMFs with equally-spaced effective indices

As stated previously, since mode coupling mediated by LPGs is a coherent, phase-matched process, a different grating is required for each pair of modes. The only way to couple all mode groups using only one uniform LPG with a fixed grating period is to ensure that the effective indices of the mode groups are designed to be nearly equally spaced. Similar to parabolic quantum wells that allow equally-spaced energy levels, GRIN fibers with a parabolic index distribution are expected to support mode groups with nearly equally-spaced effective indices. For a parabolic GRIN fiber with a core index distribution satisfying ${n}^{2}={n}_{1}^{2}-2{n}_{1}^{2}{\rm{\Delta }}{(r/a)}^{2}$ (where ${\rm{\Delta }}={n}_{1}^{2}-{n}_{2}^{2}/2{n}_{1}^{2}$, *n*_1_ and *n*_2_ are the refractive indices of the core and cladding, *r* is the radial position, and *a* is the core radius), the amplitude of the electric field ${\rm{{\rm E}}}=E({\rm{x}},{\rm{y}}){\rm{e}}{\rm{x}}{\rm{p}}(-{\rm{j}}\beta {\rm{z}})$ of a guided mode in the core satisfies^13^:

1 $$-(\frac{{\partial }^{2}}{\partial {x}^{2}}+\frac{{\partial }^{2}}{\partial {y}^{2}})E+\frac{2{k}_{1}^{2}{\rm{\Delta }}}{{a}^{2}}({x}^{2}+{y}^{2})E=({k}_{1}^{2}-{\beta }^{2})E.$$

where *k*_0_ is the free-space wavenumber, *β* is the propagation constant of the guided mode, and ${k}_{1}={k}_{0}{n}_{1}$. It is in fact a differential equation for isotropic 2D harmonic oscillators with known Hermite-Gaussian mode solutions^14^. This eigenvalue problem, assuming that the fields completely vanish in the cladding, admits standard solutions given by

2 $${k}_{1}^{2}-{\beta }^{2}=2\sqrt{\frac{2{k}_{1}^{2}{\rm{\Delta }}}{{a}^{2}}}({m}_{x}+{m}_{y}+1).$$

where ${m}_{x}=0,1,2\cdots$, and ${m}_{y}=0,1,2\cdots$ are non-negative integers representing the orders of the modes. So, the propagation constants

3 $$\beta =\sqrt{{k}_{1}^{2}-{k}_{1}\frac{2\sqrt{2{\rm{\Delta }}}}{a}({m}_{x}+{m}_{y}+1)}\approx {k}_{1}-\frac{\sqrt{2{\rm{\Delta }}}}{a}({m}_{x}+{m}_{y}+1)$$

of successive mode groups are nearly equally spaced. The design of FMFs with equally-spaced effective indices is scalable to a larger number of mode groups. As the number of mode groups grows in a parabolic GRIN fiber, the condition for equally-spaced effective indices becomes better satisfied since most of the modes are well confined.

**Figure 1:**
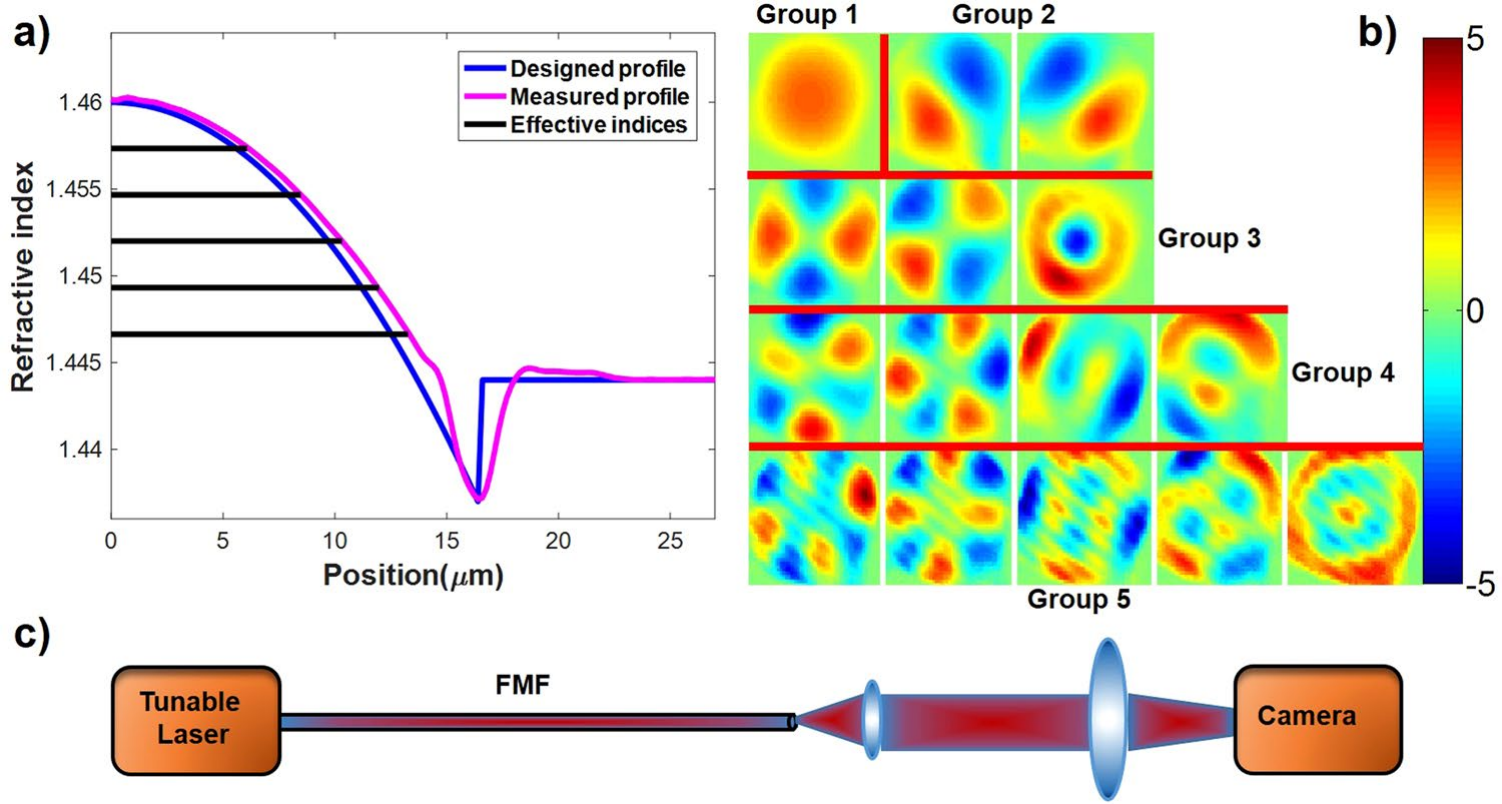
(**a**) Designed and measured index profiles of FMF, superimposed with calculated effective indices of the 5 mode groups of the measured index profile. (**b**) Mode profiles measured using the S^2^ method. (**c**) S^2^ experiment setup.

In reality, evanescent fields do exist in the cladding. So, we adopt a nearly parabolic index profile for the core with a low-index trench in order to strongly confine the guided mode to the core. An optimized design is represented by the blue curve in Fig. 1(a). We fabricated a GRIN fiber according to this design. The actual index profile of the FMF is represented by the magenta curve in Fig. 1(a), along with the calculated effective indices of modes as black lines. With ${n}_{1}=1.46,\,{\rm{\Delta }}=0.0109,a=16.4\,\mu m,{n}_{trench}=1.437$, the five mode groups (9 LP modes) at 1550 nm have almost the same effective-index differences ranging from 2.54 × 10^−3^ to 2.55 × 10^−3^ between successive mode groups for reasons explained above^6^. To characterize mode profiles of different mode groups, as shown in Fig. 1(b), we employed the spatially- and spectrally-resolved imaging (S^2^) method^15,16^ as shown in Fig. 1(c), see details in the Methods section.

## Mode coupling and GDS reduction

**Figure 2:**
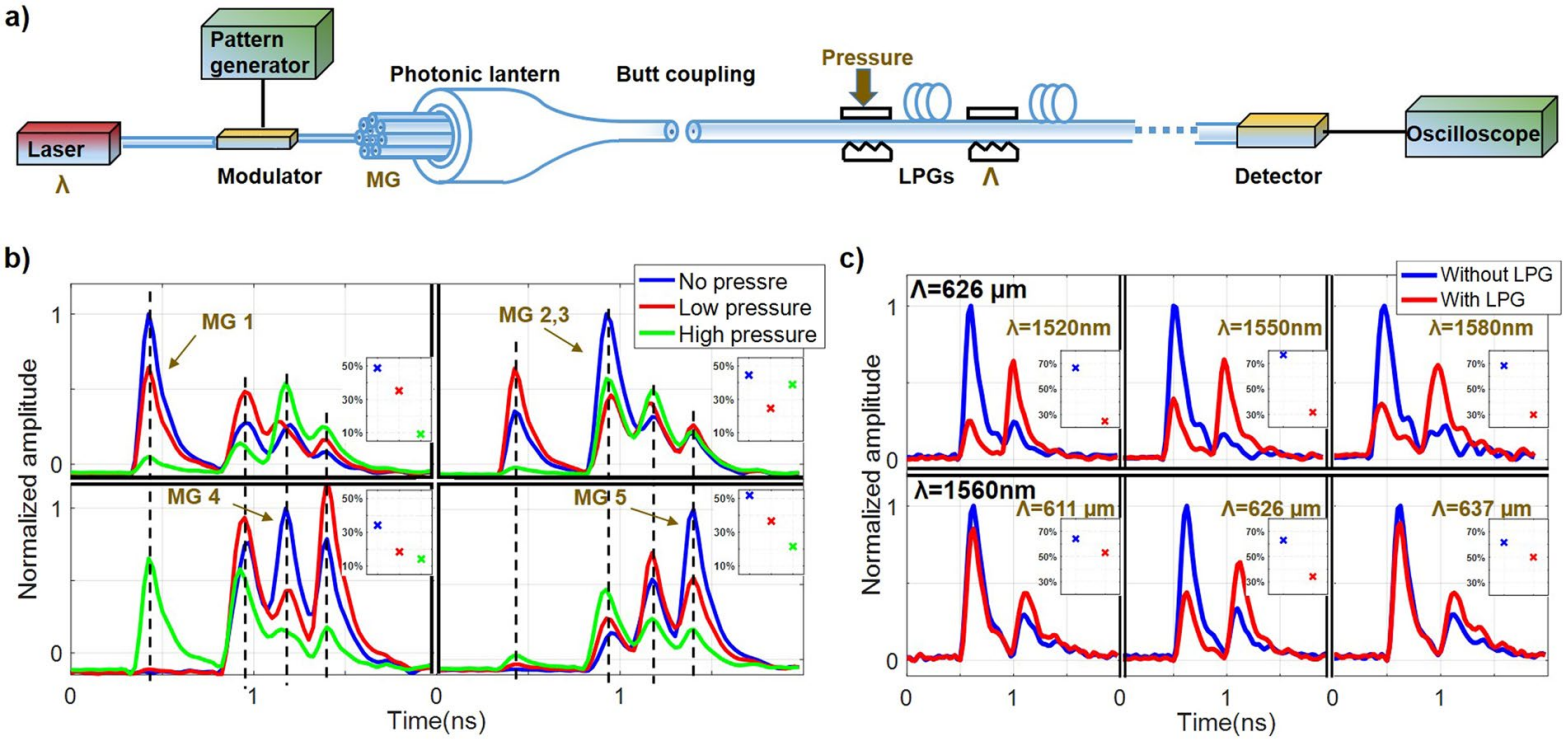
(**a**) Experimental setup for measuring the impulse response. λ: wavelength of tunable laser, Λ: period of LPGs, MG: mode group. (**b**) Measured impulse response waveforms with different pressures, when the main power is initially in MG 1, MG 2 and 3, MG 4, MG 5, respectively. (**c**) Measured impulse response waveforms with and without the LPG for different wavelengths, and for different grating periods. The insets in (**b**) and (**c**) show the percentage of power in the dominant input MG for different pressures.

We first demonstrate enhanced mode coupling among all 9 LP modes using a uniform single-period LPG. Subsequently, we use a series of such LPGs to reduce GDS. Fig. 2(a) is the experimental setup for demonstrating strong mode coupling and the reduction of GDS. The mechanical LPG consists of a lower replaceable plate with gratings cut into it and an upper flat steel plate over which an adjustable screw is used to apply pressure to the fiber sandwiched between these two plates^17,18^. This mechanical grating has the same level of uniformity as LPGs fabricated using the arc method^18^. The relative angle between the fiber and the LPG can be adjusted to change the effective grating period. Details of the experimental procedures are contained in the Methods section.

To investigate mode coupling, a single uniform mechanical LPG was applied at the beginning of the FMF. Because of the relatively large effective index difference between mode groups, coupling between mode groups is negligible without the LPG. The PL was used for selective excitation of a dominant mode group when no pressure is applied to the LPG. After applying pressure on the LPG, new modal contents are generated from mode coupling mediated by the LPG. Modal dispersion in the GRIN FMF was exploited to separate different modal groups in the time domain. Therefore, the differences in the powers of each mode group in the impulse responses of the GRIN FMF with and without pressure on the LPG can be used to characterize mode coupling mediated by the LPG. It should be pointed out that, even though the FMF has equally-spaced effective indices, the group indices are, in general, not equally spaced. It turns out that the modal dispersion between the second and third group is rather small for this particular GRIN FMF, so these two mode groups are lumped together. Figure 2(b) demonstrates that a single uniform LPG can indeed induce mode coupling between all mode groups. Taking the top left plot as an example, when no pressure was applied on the mechanical LPG, there was one main peak in the impulse response, representing that the power was mainly in the LP_01_ mode. When pressure was applied on the LPG, more modes/peaks appeared in the waveform, signifying that the power in the LP_01_ mode had been coupled into other mode groups. The red and green lines show the effect of mode coupling as the pressure on the mechanical LPG was successively increased. The inset shows the percentage of power in the dominant input LP_01_ mode for different pressures. It is observed that mode coupling increases with applied pressure. The rest of the plots in Fig. 2(b) show the impulse response waveforms when the initial power was mainly in other mode groups. They indicate that power in each mode/peak can always be coupled into not only its neighboring modes but also next-to-neighbor modes with one LPG. Similarly, the insets show the percentage of power in the dominant input MG for different pressures. When the dominant input is in MGs 2 and 3, the percentage of power in these two groups did not change monotonically with pressure, likely because the power in MG 1 is coupled back into MG 2. It can be concluded that a uniform single-period LPG can couple all the LP modes of the 5 mode groups.

The use of parabolic GRIN fiber also allows the uniform LPGs to achieve strong mode coupling for a broad range of wavelengths. The phase matching condition for coupling between two modes is^19^

4 $$\frac{\pi {\rm{\Delta }}n}{\lambda }=\frac{\pi }{{\rm{\Lambda }}}.$$

where Δ*n* is the effective index difference between the two modes, *λ* is the wavelength in free-space, and Λ is the grating period. To evaluate the bandwidth of coupling due to the LPG, effective indices of LP_01_ and LP_11_ at different wavelengths and subsequently, the left-hand side of Eq. (4), ${A}_{\lambda }=\pi {\rm{\Delta }}{n}_{\lambda }/\lambda$, were calculated. Taking both material dispersion and waveguide dispersion into consideration, the difference in *A*_*λ*_ at 1520 nm and 1580 nm is $r={A}_{1580}/{A}_{1520}-1={\rm{3}}\times {{\rm{10}}}^{-4}$, which is small enough to maintain phase matching condition with this wavelength range as shown in the top row of Fig. 2(c). The top three plots in Fig. 2(c) show that the impulse response waveforms are similar at three different wavelengths, signifying efficient, broadband mode coupling even with a fixed grating. The insets verify that mode coupling is efficient over a broadband. To verify that the broadband coupling was due to the grating rather than simply the applied pressure, the grating period was tuned to observe the change in the impulse-response waveform. As can be seen from the bottom three plots and the three insets in Fig. 2(c), mode coupling is efficient only for certain grating periods that satisfy the phase-matching condition. The grating period only affects the right-hand side ${B}_{\Lambda }=\pi /{\rm{\Lambda }}$ of Eq. (4), and the relative difference between the minimum and maximum effective periods is $r={B}_{611}/{B}_{637}-1=4.3\times {{\rm{10}}}^{-2}$ large enough to annihilate the phase matching condition as shown in the bottom row of Fig. 2(c). The total insertion loss (IL) when the effective grating period doesn’t satisfy the phase matching condition for the guided modes was measured, using the setup in Fig. 2, to be less than 0.06 dB. The loss can be considered as the extrinsic non-resonant microbending loss of the LPG.

One of the strengths of the method for enhanced mode coupling proposed here is its scalability to a larger number of modes, including modes with high azimuthal numbers. It should be recognized that what is required is for the mode with a high azimuthal number $M$ (with azimuthal dependence ${e}^{jM\phi }$) to couple to its neighbor having an azimuthal number M + 1 or M − 1 (with azimuthal dependence ${e}^{j(M+1)\phi }$ or ${{\rm{e}}}^{j(M-1)\phi }$). The asymmetry in the grating having a component ${{\rm{e}}}^{\pm j\phi }$, required to couple modes with a high or low azimuthal number to its neighbor is exactly the same, and independent of $M$.

**Figure 3:**
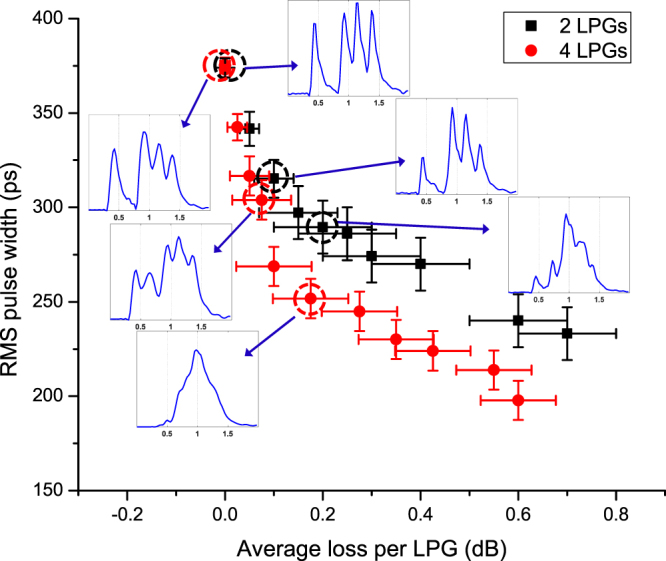
RMS pulse width as a function of average loss per LPG for the cases in which two and four LPGs were used along the 4.3 km FMF. Insets are waveforms corresponding to different losses/pressures for 2 LPGs or 4 LPGs. Horizontal and vertical error bars are calculated from the standard deviation of RMS pulse widths and losses measured under the same condition.

We then demonstrate the reduction of GDS using multiple LPGs distributed along the 4.3 km FMF. The lateral offset between the PL and FMF was adjusted to excite all modes at different group delays (GDs) with almost equal power. For each waveform measured under different pressure/mode-coupling efficiency, the RMS pulse width representing the GDS of the FMF was calculated using the following formula^13^

5 $$\sigma =\sqrt{\langle {t}^{2}\rangle -{\langle t\rangle }^{2}}$$

where $\langle {t}^{n}\rangle =\frac{1}{N}{\int }_{-\infty }^{\infty }{t}^{n}I(t)dt$, $N={\int }_{-\infty }^{\infty }I(t)dt$, and *I*(*t*) is the measured intensity waveform. Meanwhile, for each applied force, the loss induced by the LPGs was also measured. The RMS widths as functions of the measured loss induced by two or four LPGs are shown in Fig. 3. The insets are the impulse response waveforms at different losses/pressures. At low pressure (low loss), the optical power remained evenly distributed among the 4 peaks. When a strong force was applied on the grating plate, 4 peaks in the impulse response merged into an almost symmetric single peak centered at the average group delay. The RMS width decreased as the average loss/applied pressure increased. As expected, when four LPGs were used along the FMF, the GDS was reduced further compared with using only two LPGs, because GDS was accumulated over a shorter distance before modes are scrambled. Theoretically, the RMS width is proportional to $\sqrt{N}$(*N* is the number of LPGs)^5^ when the length of fiber between two LPGs is the same. Thus when the total length is the same, the RMS width is proportional to $1/\sqrt{N}$. The fluctuations in RMS width and loss are due to environmental changes such as temperature and fiber deformation.

## Reducing intrinsic loss and MDL

**Figure 4:**
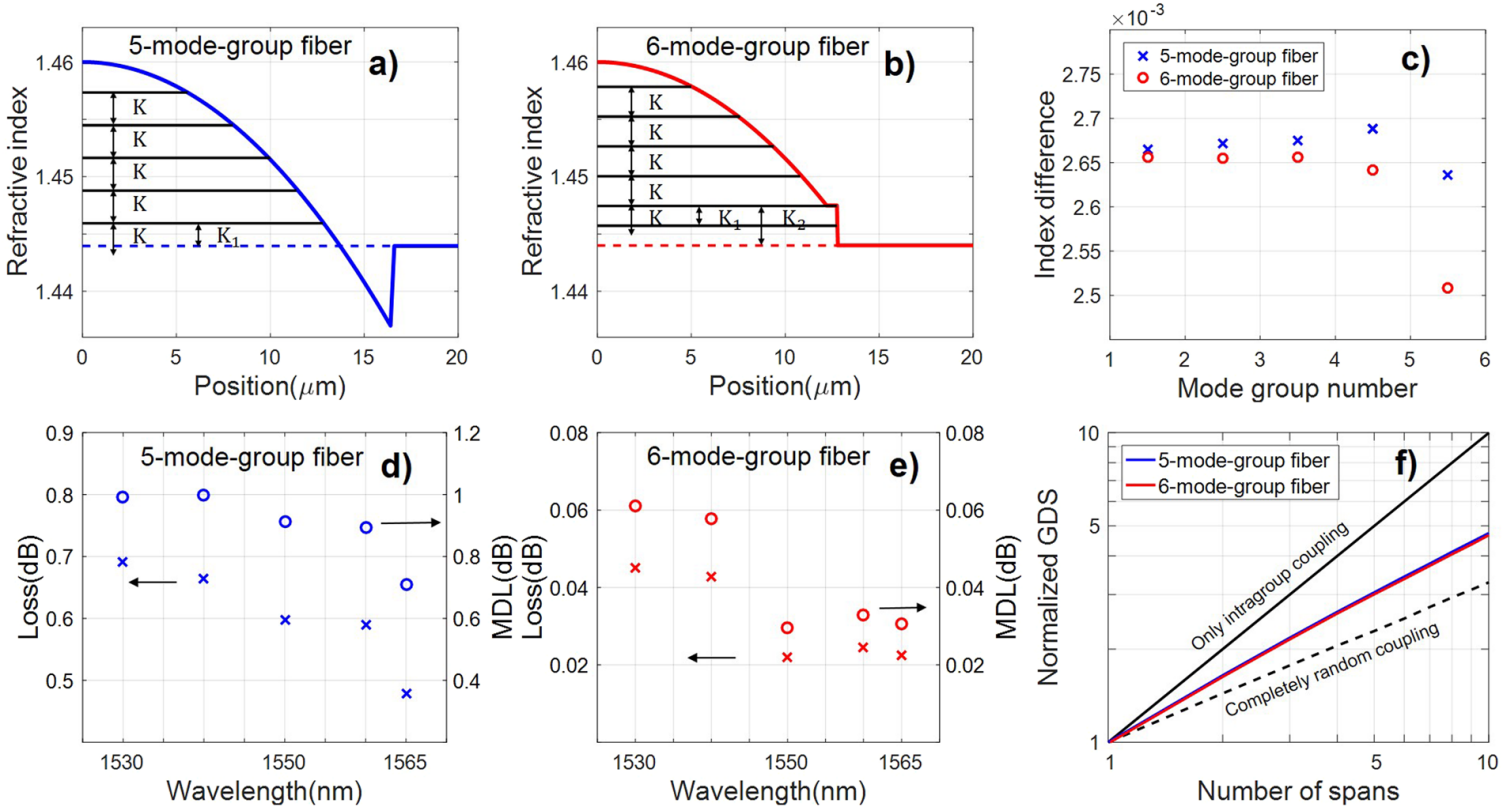
(**a**,**b**) Index profiles of the 5-mode-group fiber and the 6-mode-group fiber. Black lines represent effective indices of each mode group. K is average refractive index difference between two neighboring mode groups that are nearly equally spaced, ${K}_{1} < K < {K}_{2}$. (**c**) Index differences between mode groups in the 5-mode-group fiber and the 6-mode-group fiber, respectively. The last blue point represents the index difference between the highest-order core mode group and the cladding index. (**d)**, (**e**) IL and MDL vs. wavelength for LPGs written in the 5-mode-group fiber and the 6-mode-group fiber, respectively. (**f**) Normalized GDSs as functions of the number of spans using LPGs written in the 5-mode-group fiber and the 6-mode-group fiber, compared with the cases of intragroup coupling and completely random coupling.

We now turn our attention to reducing the intrinsic loss and MDL. We use the RMS of log-unit MDL here, which is the statistically important parameter for characterizing MDL in the strong coupling regime^20^. Figure 3 shows that significant mode mixing can be achieved using LPGs, accompanied by an average loss of 0.6 dB. It is desirable to further reduce this loss. The main source of the loss is due to the seemingly unavoidable power transfer from the highest-order mode group into cladding modes. This also means that the resulting MDL is large. In order to alleviate this problem, we propose the use of a specially designed fiber for the LPG, which supports at least one more mode group than that supported by the fiber for transmission. The rationale is explained in Fig. 4 by comparing two different fibers used in the grating section for the same transmission fiber that supports five mode groups. The fiber in Fig. 4(a) is a trench-assisted GRIN fiber, and it supports five mode groups. The index profile is adjusted to make the effective indices of the five mode groups equally spaced to ensure efficient mode coupling, and the use of a trench was found to be necessary. The fiber in Fig. 4(b) is a GRIN fiber with a pedestal at the core-cladding boundary, and it supports six mode groups, one more group than that supported by the transmission fiber. The index profile is adjusted to make the effective indices of the first five mode groups equally spaced, and the effective index difference between the fifth and sixth mode groups much smaller than the average index difference between the first five mode groups. Eliminating the trench and adding the index pedestal was found to be necessary. We used the finite-element method (FEM) for the mode solver. The two fibers in Fig. 4(a) and (b) have the same core/cladding indices but different radius values of 16.4 µm and 12.75 µm and *α* values of 2.0 and 1.986, respectively. The pedestal has a width and height of 0.55 µm and 0.0035, respectively.

The black lines in Fig. 4(a) and (b) represent the effective indices of mode groups in these two fibers. It can be seen that the effective index difference between the highest-order mode group and the cladding index is always smaller than the average index difference between the neighboring core mode groups for both index profiles. Figure 4(c) plots the effective index differences between neighboring mode groups. The last blue point in Fig. 4(c) represents the index difference between the highest core mode group and the cladding index in the 5-mode-group fiber. So, when a LPG phase matched for core mode coupling is applied on the 5-mode-group FMF, the highest-order mode group would be easily coupled to some cladding modes, incurring a large intrinsic loss.

This seemingly unavoidable intrinsic loss can be eliminated using the fiber in Fig. 4(b) that supports one more mode group than the 5-mode-group transmission fiber. As can be seen in Fig. 4(b), the index difference between the last two mode groups (K_1_ = 2.51 × 10^−3^) is much smaller than the average effective index difference between successive core mode groups (K = 2.65 × 10^−3^) as shown in Fig. 4(c), while the effective index difference between the second highest-order mode group and the cladding index (K_2_ = 2.73 × 10^−3^) is much larger than K. In this case, it will be inefficient for the first 5 mode groups to couple into either the highest-order mode group or the cladding modes. So when signals are contained in the first 5 mode groups in the 6-group FMF, the intrinsic loss of the LPG can be significantly reduced.

The reduced IL and MDL of the proposed method has been verified by numerical simulations. Our method relies on the properties of the FMF in which the grating is applied, more so than the particular types of gratings that are used. In our simulations here we assume tilted index gratings rather than mechanical gratings were used. We assume that the MDM signal is carried on a transmission fiber that supports 5 mode groups and LPGs are used periodically to enhance mode coupling. LPGs written in a 5-mode-group GRIN FMF and a 6-mode-group GRIN FMF, as described above, are compared. The GDSs for these two cases are computed from the eigenvalues of the group delay operators, which, in turn, are computed from the transfer matrix of the fiber link^21^ (see details in the Methods section).

To provide a fair comparison of IL and MDL, we adjusted the parameters of the LPGs for these two cases so that the reductions of GDS for these two cases are statistically identical. To do so, we plot the ensemble average of the standard deviations of the group delays, normalized by the group delay of one span for 100 instances of the random intragroup coupling matrices and the span lengths as a function of the number of spans. The nearly identical GDSs for these two cases, as shown in Fig. 4(f), were achieved with a grating length of 3.5 cm, tilt angle of ${85}^{o}$, and index contrasts of 5.5 × 10^−5^ and 6 × 10^−5^ for the index LPGs written in the 5-mode-group GRIN FMF and a 6-mode-group GRIN FMF, respectively. Tilting is necessary because different spatial modes are orthogonal to each other, and there would be no coupling between different modes without tilting. The GDSs for these two cases increase approximately with the square-root of the number of spans (or propagation length) due to strong mode coupling mediated by both types of LPGs. The GDSs as a function of the number of spans for the case of intragroup coupling only and completely random coupling among all modes are also shown for comparison.

From the coupling matrices of the LPGs used for Fig. 4(f), we use singular-value decomposition to compute the IL and MDL as functions of wavelength for the two types of LPGs^20^. Using LPGs in a FMF with the same number (5) of mode groups as the transmission fiber, the IL and MDL are in the 0.6 dB and 1 dB range, as shown in Fig. 4(d). On the other hand, using LPGs in a FMF with one more mode group than the transmission fiber, the IL and MDL are reduced to below 0.05 and 0.06 dB respectively, as shown in Fig. 4(e). As can be seen, using LPGs in a FMF supporting one more mode group than the transmission fiber significantly reduces the loss and MDL over the entire C band.

## Conclusions

In conclusion, we propose and experimentally demonstrate the reduction of GDS using LPGs with very low insertion loss and mode-dependent loss. By designing a FMF with equally-spaced effective indices on which the LPG is applied, all mode groups in the FMF can be efficiently coupled using just one uniform LPG with a fixed grating period, instead of a different LPG for each mode group pair. In addition, by applying the LPG in a FMF that supports at least one more mode group than the transmission fiber, insertion loss and mode-dependent loss due to coupling from the core mode to the cladding mode can be largely suppressed. These strategies lead to the lowest intrinsic and extrinsic losses as well as mode-dependent loss to date, for inducing strong mode coupling using LPGs. Furthermore, we have verified that low-loss strong mode coupling could be achieved over a broad range of wavelengths. By periodically applying these LPGs along the transmission fiber, GDS increases as the square root of the transmission distance, rather than linearly without strong mode coupling. These results illustrate that simple LPGs can serve as a practical tool to reduce the GDS in FMFs, thus overcoming the MIMO DSP complexity issue, which is one of the most critical challenges for the practical implementation of mode-division multiplexed systems.

## Methods

In the S^2^ method of mode characterization, a camera was used to record output images of the FMF when light of different wavelengths was launched. By using principal component analysis (PCA) and independent component analysis (ICA)^15,16^, we acquired profiles of all 9 LP modes in Fig. 1(b).

We used the setup in Fig. 2(a) to demonstrate enhanced coupling and the reduction of GDS. A data pattern from the pattern generator was used to modulate the light from a laser. The pattern generator produced a short pulse, which consisted of one bit 1 and a long series of bit 0 s. After modulation, an impulse of light was launched into one of the input fibers of the photonic lantern (PL), which was connected with the GRIN FMF by butt coupling. The 15-mode mode-selective PL made in-house, although being state-of-the-art, cannot guarantee exciting exactly one mode group at a time. After propagation through the FMF, the signal was detected by a multimode InGaAs PIN + TIA receiver. The receiver was connected to the oscilloscope to record the impulse response waveforms. The pulse generator is Hewlett-Packard 70841B with 3 G bandwidth, the modulator is OKI EAM OM5753C30B with 30 G bandwidth, the receiver is Discovery R402 PIN-TIA with 10 GHz bandwidth, and the oscilloscope is Agilent Infiniium DSO81204A with 12 G bandwidth.

In the simulations to demonstrate the reduction of loss using LPGs written on FMFs that support one more mode group, we obtain the transfer matrix of the link by multiplying the propagation matrix, including the effect of random intragroup coupling, of each transmission fiber span and the coupling matrix of each LPG. Completely random coupling between degenerate modes in a mode group, represented by a random unitary matrix, is assumed as this indeed occurs in real fibers. An extra length of fiber uniformly distributed between ± 1 m is added to each span to account for the imprecise positions of the LPGs. To obtain the coupling matrix of the LPGs, we first compute the mode profiles of core modes and cladding modes of the GRIN FMFs, and then the coupling coefficients among all modes. Subsequently, we use the coupled-mode equations (CMEs) to calculate the coupling matrix of the LPGs^22–24^ among core modes as well as cladding modes. To ensure the calculated loss is accurate, 4 cladding mode groups are included.

### Data availability

All data generated or analysed during this study are included in this published article.
